# Increased Intragenic *IGF2* Methylation is Associated with Repression of Insulator Activity and Elevated Expression in Serous Ovarian Carcinoma

**DOI:** 10.3389/fonc.2013.00131

**Published:** 2013-05-24

**Authors:** Zhiqing Huang, Susan K. Murphy

**Affiliations:** ^1^Epigenetics Research Laboratory, Division of Gynecologic Oncology, Department of Obstetrics and Gynecology, Duke University Medical Center, Durham, NC, USA; ^2^Department of Pathology, Duke University Medical Center, Durham, NC, USA

**Keywords:** insulin-like growth factor 2, epithelial ovarian cancer, DNA methylation, chromatin immunoprecipitation, CTCF, spermatozoa, germline methylation, luciferase reporter assay

## Abstract

Overexpression of insulin-like growth factor-II (IGF2) is a prominent characteristic of many epithelial ovarian malignancies. *IGF2* imprinting and transcription are regulated in part through DNA methylation, which in turn regulates binding of the insulator protein CTCF within the *IGF2/H19* imprint center. We have shown that *IGF2* overexpression in ovarian cancer is associated with hypermethylation of CTCF binding sites within the *IGF2/H19* imprint center. The aim of this study was to investigate the methylation and binding capacity of a novel putative CTCF binding motif located intragenic to *IGF2* and determine how this relates to *IGF2* expression. Among 35 primary serous epithelial ovarian cancer specimens, methylation of two CpGs, including one within the core binding motif and another adjacent to this motif, was higher in the 18 cancers with elevated *IGF2* expression versus 10 with low expression (average 68.2 versus 38.5%; *p* < 0.0001). We also found that the CpG site within the CTCF binding motif is hypermethylated in male gametes (>92%; average 93.2%; *N* = 16). We confirmed binding of CTCF to this region in ovarian cancer cells, as well as the paralog of CTCF, Brother Of the Regulator of Imprinted Sites (BORIS), which is frequently overexpressed in cancers. The unmethylated CTCF binding motif has insulator activity in cells that express CTCF or BORIS, but not in cells that express both CTCF and BORIS. These intragenic CpG dinucleotides therefore comprise a novel paternal germline imprint mark and are located in a binding motif for the insulator protein CTCF. Methylation of the CpG dinucleotides is positively correlated with *IGF2* transcription, indicating that increased methylation represses insulator function. These combined results suggest that methylation and CTCF binding at this region play important roles in regulating the level of *IGF2* transcription. Our data have revealed a novel epigenetic regulatory element within the *IGF2/H19* imprinted domain that is highly relevant to aberrant *IGF2* expression in ovarian malignancies.

## Introduction

Insulin-like growth factor-II (*IGF2*) encodes a potent autocrine and paracrine mitogen that is important to early growth and development (Hoyo et al., 2012; St-Pierre et al., 2012), but is also often deregulated in cancers, including colon (Cui et al., 2003) and ovarian cancers (Sayer et al., 2005; Lu et al., 2006; Murphy et al., 2006; Dammann et al., 2010; Qian et al., 2011) where it has been shown to modulate taxol response (Huang et al., 2010). The *H19* gene is located adjacent to *IGF2* and produces a long non-coding RNA molecule whose function is not completely understood. Both genes are regulated by genomic imprinting, resulting in the production of *IGF2* transcripts from the paternally derived chromosome and *H19* RNAs from the maternally derived chromosome. Epigenetic marks dictate this pattern of expression, and are established during gametogenesis in a process that involves the differential marking of the parental chromosomes with DNA methylation. These marks are then “read” such that expression occurs from only one of the two parental alleles, and this pattern of expression and epigenetic marking is stably transmitted throughout somatic cell division.

Prior studies have determined that a region upstream of the *H19* promoter is methylated on the paternally derived chromosome, while the maternally derived chromosome is hypomethylated. Within this differentially methylated domain, referred to as the imprint control region (ICR), are multiple binding sites for the CCCTC binding factor (CTCF) protein. CTCF is a highly conserved zinc finger protein that binds to genomic DNA at numerous sites throughout the genome (Sanyal et al., 2012; Wang et al., 2012) and has played a key role in the evolutionary diversity of metazoans (Heger et al., 2012). One of the functions of CTCF is the remodeling of chromatin structure to form insulator elements (Filippova, 2008), and CTCF has been shown to co-localize with cohesin in the control of chromatin architecture and gene regulation (Lee and Iyer, 2012). The importance of CTCF binding to genomic imprinting was demonstrated by several groups who showed that CTCF binds the unmethylated maternal chromosome, upstream of the promoter region of the *H19* gene within the ICR. CTCF binding prevents enhancers downstream from *H19* from activating *IGF2* on the maternal chromosome, while the methylation present on the paternally derived chromosome prevents CTCF binding. *IGF2* transcription is thus positively influenced by the unencumbered *cis*-acting enhancer elements (Wolffe, 2000).

Prior studies, including our own, have identified a consensus binding motif, 5′-CCGCNNGGNGNC-3′ for the CTCF protein (Wylie et al., 2000; Rosa et al., 2005). We identified a sequence within *IGF2* intron 3 that matches this consensus motif, and our preliminary analysis showed a strong relationship between methylation of this putative binding site and expression of *IGF2* in ovarian cancer tissues. Herein we examined the methylation status of this site, including parental origin, and show that this novel consensus sequence binds to CTCF and mechanistically functions as an insulator element in ovarian cancer cells.

## Materials and Methods

### Specimens

De-identified primary ovarian cancer tissues for this study were obtained from the Duke Gynecologic Oncology Tissue Bank (DCOTB) under a protocol approved by the Duke University Institutional Review Board. The DCOTB collects and banks specimens for research purposes following acquisition of informed written consent from patients undergoing surgery for epithelial ovarian cancer under a separate protocol approved by the Duke University Institutional Review Board. Surgical specimens were processed immediately after removal from the patient and stored at −80°C. Human spermatozoa were from the Duke Division of Reproductive Endocrinology and Fertility and used under a protocol approved by the Duke Institutional Review Board. Conceptal tissues were provided as de-identified specimens by the Laboratory of Developmental Biology at the University of Washington (supported by NIH Award Number 5R24HD000836 from the Eunice Kennedy Shriver National Institute of Child Health and Human Development).

Cell lines were from a collection maintained by the Duke Division of Gynecologic Oncology. The genetic authenticity of the cells used in these studies was determined using microsatellite marker analysis at the University of Colorado at Denver (Korch et al., 2012). Cells were grown in RPMI1640 medium (Invitrogen) supplemented with 10% fetal bovine serum (Invitrogen) and penicillin–streptomycin (Invitrogen, Carlsbad, CA, USA) in a humidified chamber at 37°C with 5% atmospheric CO_2_.

### DNA extraction

Tissue culture cells were washed with 1× PBS and collected after they reached 70–80% confluence. Frozen tumor tissues were homogenized using a FastPrep SP120 Homogenizer (Bio101 Thermo Savant; Logan, UT) and collected into microcentrifuge tubes. DNA was extracted using Gentra Puregene Reagents with protocols provided by the manufacturer specific to the sample type (Qiagen, Valencia, CA, USA). Quality and quantity of extracted DNA was evaluated using a Nanodrop 1000 spectrophotometer (Thermo Scientific; Wilmington, DE, USA). Aliquots were prepared and stored at −80°C. The A260/A280 absorbance ratios were >1.8 for all DNA used.

### Methylation analysis

Two methods were used for CpG methylation analysis of the *IGF2* intragenic region. Quantitative radiolabeled bisulfite sequencing was used for frozen ovarian cancer tissues. Because of the manufacturer’s permanent discontinuation of the radiolabeled isotope used for quantitative bisulfite sequencing, bisulfite pyrosequencing was used for all other analyses.

#### Bisulfite sequencing

Genomic DNA (1 μg) was modified with sodium bisulfite as previously described (Murphy et al., 2003) and eluted in 25 μl of nuclease-free water. Forty nanograms of the bisulfite modified DNA (assuming complete recovery) was used in a 25 μl PCR reaction that included 3 mM MgCl_2_, 1 mM each dNTP, and 0.4 μM each primer with 1.25 U Platinum Taq (Invitrogen; Carlsbad, CA, USA). A 290 bp PCR product specific to intron 3 of *IGF2* was generated with primers listed in Table 1. PCR conditions were 94°C for 3 min, 35 cycles of 94°C for 30 s, 62°C for 30 s, 72°C for 45 s, with a final 5 min extension at 72°C. The amplicons were resolved by agarose gel electrophoresis, excised, and purified using Sigma GenElute Spin columns (Sigma Aldrich). Bisulfite sequencing was performed using the Radiolabeled Terminator Cycle Sequencing Kit (USB Corporation) according to the manufacturer’s directions. Sequencing products were resolved by denaturing polyacrylamide gel electrophoresis and the gels were dried under vacuum followed by exposure to a storage phosphor screen. Methylation was quantified using the Molecular Dynamics Storm Phosphorimaging System and ImageQuant software (GE Healthcare; Pittsburgh, PA, USA). The percent cytosine methylation was calculated as follows: [*C*^M^/(*C*^U^ + *C*^M^)] × 100, where *C*^M^ is the measurement for cytosine at a given CpG dinucleotide position and *C*^U^ is the measurement for thymine at the same position of the cytosine base (which reflects unmethylated cytosine after bisulfite conversion and PCR amplification).

**Table 1:** **Oligonucleotide primers**.

Primer	Application	Sequence (5′–3′)
Int3-IGF2-BS-F	Bisulfite sequencing	GTTTTTTTGGTTGTGGGATTAAGAG
Int3-IGF2-BS-R	Bisulfite sequencing	AACCACACAACAAACAAAAAAATC
Int3-IGF2-IR-seq	Bisulfite sequencing	CAAACCACCCCTACCCTC
Int3-CTCF3-PS-F	Pyrosequencing	TTGGGAAGTTTTTGTTTGTTAGT
Int3-CTCF3-PS-R	Pyrosequencing	Biotin-ACCCTCAAACCAAACCCTAA
Int3-CTCF3-PS-seq	Pyrosequencing	GGAAGTTTTTGTTTGTTAGT
Int3-CBS-chip-F	Chromatin IP	ACTCACCTCCCCTCTCAC
Int3-CBS-chip-R	Chromatin IP	AGCAAACCACCCCTGCC
MYC-chip-F	Chromatin IP	CCTGAAAGAATAACAAGGAGGTGGCTGGAAACTTG
MYC-chip-R	Chromatin IP	GCAAATTACTCCTGCCTCCAGGCCTTTG
*Nhe*I-CTCF-F2	Luciferase reporter	ATATTGCTAGCCACATCCCACACATTTTCCA
R2-CTCF-*Xho*I	Luciferase reporter	AAAGCTCGAGGAGAGAGCCGTGTTAGCAC
GAPDH-*Sma*I-F	Luciferase reporter	ATATTAGCCCGGGTGGTCTGAGGTTAAATATAGC
GAPDH-*Bgl*II-R	Luciferase reporter	TAATTGCAGATCTAAGAGACAAGAGGCAAGAAGG

#### Bisulfite pyrosequencing

Genomic DNA (800 ng) was modified with sodium bisulfite using the Zymo EZ DNA methylation kit (Zymo Research Corporation, Irvine, CA, USA). For pyrosequencing of the intron 3 region of *IGF2*, we amplified bisulfite modified DNA using 0.12 μM of each primer (all primers are listed in Table 1) in a 25 μl reaction containing 40 ng of bisulfite modified template DNA, 1.5 mM MgCl_2_ and using the PyroMark PCR Kit (Qiagen; Valencia, CA, USA). Cycling conditions were 95°C for 15 min, then 55 cycles of 94°C for 30 s, 59°C for 30 s, and 72°C for 30 s, followed by 10 min at 72°C. Pyrosequencing was performed using Pyromark Gold Q96 Reagents according to the manufacturer’s recommendations (Qiagen). The sequence analyzed includes two CpG dinucleotides; one located within the consensus CTCF binding motif and a second CpG outside the consensus motif (5′…gaccCGcagggtggctgCGtcc…3′; consensus underlined).

To validate the pyrosequencing assay, we used plasmids containing bisulfite modified methylated and unmethylated copies of the region analyzed, as previously described (Wong et al., 2006; Murphy et al., 2012). The plasmid DNAs were quantified, defined mixtures prepared, and run in quadruplicate. Pyrosequencing assay design and validation for the *IGF2* DMR was as described previously (Murphy et al., 2012).

### RNA analyses

Cells were cultured until 70–80% confluent and washed with cold PBS. Total RNA was isolated from these cells and from frozen human conceptual tissues with RNA Stat-60 reagent (Amsbio, Lake Forest, CA, USA). One microgram was reverse transcribed using Superscript II according to the manufacturer’s protocol (Invitrogen). The resulting cDNA was amplified using a TaqMan assay for *IGF2* expression (Assay ID: Hs04188276_m1) with a Rotor-Gene Q Real-Time PCR thermocycler (Qiagen). Beta-2-Microglobulin (*B2M*) (Assay ID: Hs00187842_m1) was used as a control for input. Expression values were determined using the delta delta *C*_t_ method. *IGF2* mRNA expression values from cell lines were obtained from our previously published Affymetrix U133A microarray data (Gene Expression Omnibus Accession GSE25428) and expression values were validated using quantitative real-time RT-PCR.

### Protein analysis

Western blotting was performed to determine the endogenous levels of CTCF protein in the ovarian cancer cell lines. Briefly, 30 μg of total cell lysate was obtained using cell lysis buffer from cells that were cultured to 70–80% confluence (Cell Signaling). Antibodies used were anti-CTCF (Millipore #07-729, diluted 1:2000) and anti-α-Tubulin (Millipore #05-829, diluted 1:200). The anti-mouse HRP conjugated secondary antibody (Bio-Rad; Hercules, CA, USA) was used at a dilution of 1:3000 and the proteins were detected with the ECL kit (GE Healthcare) following the manufacturer’s instructions.

### Chromatin immunoprecipitation

Chromatin Immunoprecipitation (ChIP) was performed with the ChIP-It kit (Active Motif; Carlsbad, CA, USA) using 25 μg of sheared chromatin per IP and 2 μg of each antibody. The rabbit anti-human CTCF polyclonal antibody used was from Millipore (#07-729), the anti-Brother Of the Regulator of Imprinted Sites (BORIS) antibody was from Abcam (Ab18337; Cambridge, MA, USA) as well as the rabbit IgG control antibody (ab37415). Real-time PCR was performed using SYBR green reagents (Qiagen) in an Applied Biosystems 7900HT Real-Time PCR System. Primers are listed in Table 1. The 5′-insulator site in the *c-MYC* gene constitutively binds CTCF and was used as a positive control for CTCF binding as previously described (Pugacheva et al., 2005).

### Insulator assays

The DNA fragment containing the intron 3 CTCF consensus sequence was PCR amplified using the primers listed in Table 1. The product was cloned into the pGL3-control Luciferase Reporter Vector (pGL3-P-control; Promega, Madison, WI, USA) using the *Nhe*1 and *Xho*1 restriction sites. This plasmid contains the luciferase gene under control of the SV40 promoter with SV40 enhancer sequences located downstream. The multi-cloning site is upstream of the SV40 promoter region. A sequence fragment from within the *GAPDH* locus was also used for comparative purposes (see Table 1). This fragment was cloned into the pGL3-P-control using *Sma*I and *Bgl*II restriction sites. The recombinant plasmids were confirmed by restriction digestion and by nucleotide sequencing. Constructs were named pGL3 (vector only), pGL3-CTCF, and pGL3-GAPDH. HEY, CAOV2, and OVCAR2 cells were grown to 70–80% confluence and transfected with the various pGL3 plasmid constructs using FuGENE^®^HD reagent according to the manufacturer’s protocol (Promega, Madison, WI, USA). Luciferase activity was measured 48 h and 72 h post-transfection in sextuplicate according to the protocol from the manufacturer (Promega). Luciferase activity was normalized for cell number.

### Statistical analysis

All statistical analyses were performed using Prism 5 for Macintosh (Graphpad Software, Inc.) with *p* values less than 0.05 considered significant.

## Results

### Identification of a CTCF binding motif in *IGF2* intron 3

**Figure 1:**
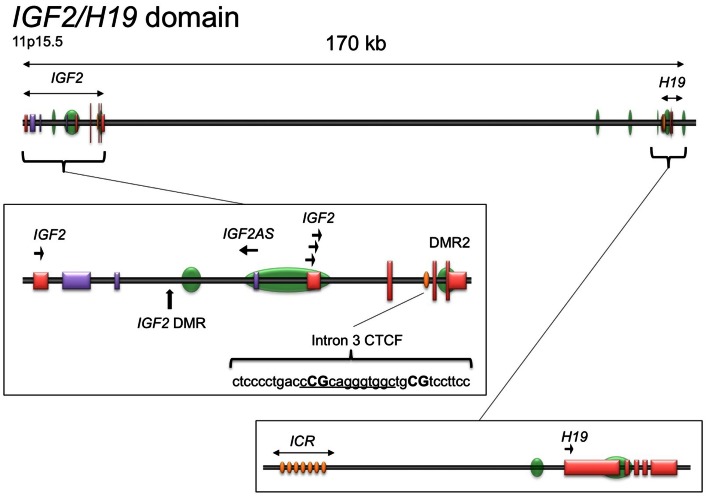
**Schematic of the *IGF2/H19* imprinted domain**. The *IGF2/H19* imprinted domain is located at chromosome 11p15.5 and encompasses ∼170 kb. The insets show the genomic structure of the *IGF2* gene (middle) and *H19* gene (bottom), adapted from the UCSC Genome Browser, GRCh37/hg19 (Kent et al., 2002). Green ovals, CpG islands; yellow ovals, CTCF binding sites; tall vertical lines/boxes, coding sequence; short vertical lines/boxes, non-coding sequence. The promoters with direction of transcription are indicated by arrows. The *IGF2* locus also produces a paternally expressed antisense transcript (*IGF2AS*), indicated by the purple lines/boxes. The *IGF2* intron 3 CTCF binding motif is shown as underlined sequence with the CpG dinucleotides analyzed herein in capital letters. DMR, differentially methylated region; ICR, imprint control region.

The genomic structure of *IGF2* is presented in Figure 1. Given the importance of CTCF binding upstream of the *H19* promoter to the imprinting and expression of *IGF2* and *H19* and the ubiquity of *IGF2* overexpression in serous epithelial ovarian cancer (Murphy et al., 2006), we wished to determine if there are other CTCF binding sites within the *IGF2/H19* imprinted domain that contribute to regulation of *IGF2* in this disease. By searching the genomic sequence, we identified a binding motif within *IGF2* intron 3 having sequence matching that of the consensus motif for CTCF binding (5′-CCGCNNGGNGGC-3′) (Wylie et al., 2000; Rosa et al., 2005). Remarkably, the position of this motif is ∼ 350 bp downstream of an inverted repeat element within the same intron, in a region that was identified as being conserved among mammals that exhibit imprinted *IGF2* expression (Weidman et al., 2004). The inverted repeat has subsequently been found to encode a microRNA, MIR483 (NR_030158.1) that has been implicated in angiogenesis, oncogenicity, and adipose tissue programing (Veronese et al., 2010; Qiao et al., 2011; Ferland-McCollough et al., 2012; Ma et al., 2012).

### Intragenic *IGF2* methylation correlates with expression

**Figure 2:**
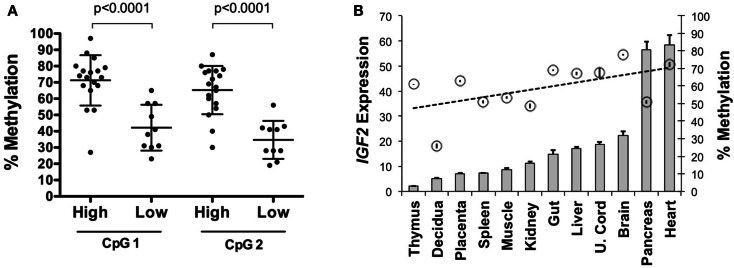
***IGF2* intragenic methylation and *IGF2* transcription**. **(A)** Methylation of the CpG within the *IGF2* intron 3 CTCF binding motif (CpG 1) and adjacent CpG (CpG 2) in invasive serous ovarian cancers with very low *IGF2* expression (Affymetrix U133A MAS5 values between 9 and 71, average = 26; labeled as “low”) compared to those with higher expression (MAS5 values between 128 and 24,056, average = 5,343; labeled as “high”). **(B)** *IGF2* expression shown as a bar graph (+SD for replicate measures; left *y* axis) as detected in tissues from a 108-day gestation human female conceptus with the corresponding level of methylation, given as a percentage, at the CpG within the intron 3 CTCF binding motif (right *y* axis, circles ± SD of replicate measures). The data are arranged from left to right in increasing order of *IGF2* expression.

To determine if *IGF2* expression levels are associated with intragenic methylation of *IGF2* at this region, we analyzed 35 primary serous epithelial OVCA specimens using quantitative radiolabeled bisulfite sequencing. We found highly variable methylation at the region adjacent to and within the *IGF2* intron 3 consensus CTCF binding motif. Methylation of two CpGs, including one within the core binding motif, was higher in 18 OVCAs with elevated *IGF2* expression versus 10 with low expression (average 68.2% versus 38.5%; *p* < 0.0001) (Figure 2A). We also examined the relationship between expression and methylation in a panel of tissues from a 108 day human female conceptus, and found that most tissues with higher methylation also had higher *IGF2* expression (gut, liver, umbilical cord, brain, pancreas and heart versus thymus, decidua, placenta, spleen, muscle, and kidney; *p* = 0.03 for *t* test between these groups; data presented in Figure 2B is by individual tissue for clarity). These results support that the methylation status of these intronic CpG dinucleotides is positively associated with *IGF2* expression and may therefore be involved in regulating levels of *IGF2*.

### Germline methylation

**Figure 3:**
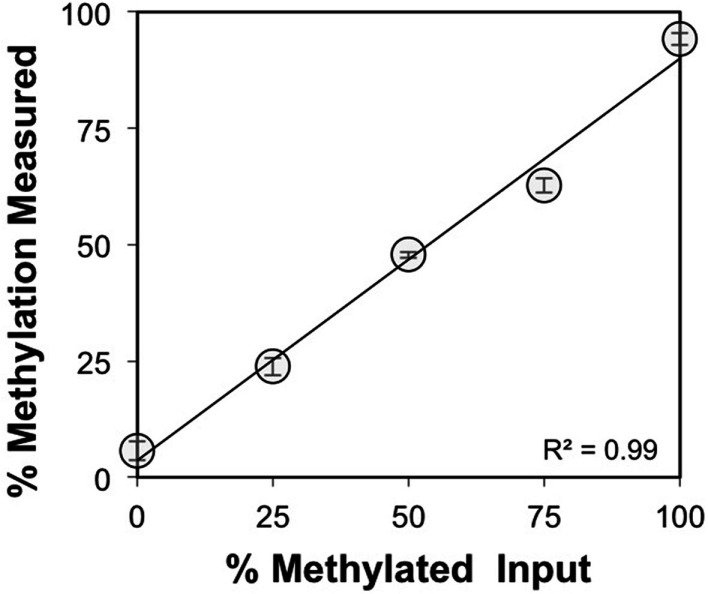
**Validation of the *IGF2* intron 3 region pyrosequencing assay**. Methylation was measured for both CpG dinucleotides within the intron 3 region under study and averaged (*y* axis, ±SD for triplicate measures) for defined mixtures of plasmids (*x* axis) containing the bisulfite modified methylated and unmethylated intron 3 region sequences.

**Figure 4:**
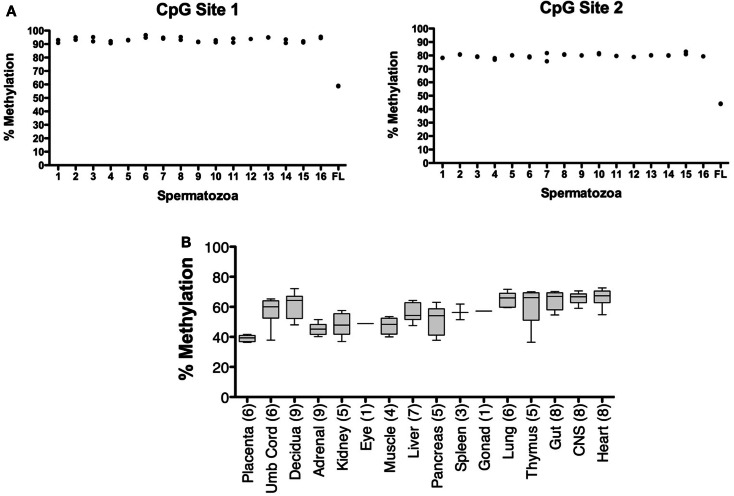
**Characterization of *IGF2* intron 3 CTCF binding motif methylation in spermatozoa and human fetal tissues**. **(A)** Measurement of % methylation (*y* axis) for both CpG dinucleotides (CpG Site 1, CpG within the CTCF binding motif; CpG Site 2, CpG adjacent to the CTCF binding motif) in 16 individual human spermatozoa specimens (1–16 on *x* axis) and in human fetal liver (FL). The results from each of two assays are shown. **(B)** Box and whisker plot showing average percent methylation for the two CpG dinucleotides in the *IGF2* intron 3 region across a panel of human conceptal tissues (boxes, 10th to 90th percentile). The number of individuals analyzed for each tissue type is indicated.

The CpG dinucleotides within the CTCF binding motifs of the *IGF2/H19* ICR are methylated on the paternal chromosome and this methylation profile is established during gametogenesis. Because the ICR and intron 3 CpGs are located greater than 100 kb apart (Figure 1), we analyzed the *IGF2* intron 3 CpG sites in 16 independent human spermatozoa specimens to determine if these CpGs are also methylated in sperm. These and subsequent analyses were performed by bisulfite pyrosequencing after validating the linear performance of the assay using defined mixtures of fully methylated and unmethylated DNAs (input versus measured methylation *R*^2^ = 0.99; Figure 3). We found that the intron 3 CpG sites exhibited high levels of methylation in sperm from all 16 individuals (Figure 4A; results from replicate measures shown). However, comparison of the two intron 3 CpGs revealed significant differences in methylation levels despite being located just 13 bp apart (CpG 1: mean 93.2%, SD = 1.7%; CpG 2: 79.7%, SD = 1.4%; paired *t* test *p* < 0.0001). Human fetal liver tissue run in parallel was 58.9 and 44.0% methylated at these CpG sites, respectively.

Analysis of human conceptual tissues from up to nine individuals showed intermediate levels of methylation at these same sites (Figure 4B; range 36.3–72.6%), suggesting that the methylation in somatic tissues may be parent-of-origin-dependent, although this was not formally determined in this study. We also analyzed the *IGF2* DMR in the same spermatozoa specimens. The *IGF2* DMR and P2–P4 promoter region have previously been reported as methylated on the maternally derived chromosome (Issa et al., 1996; Sullivan et al., 1999), although subsequent studies have found that methylation is present on the paternally derived chromosome (Murrell et al., 2008). We observed that the *IGF2* DMR is highly methylated in sperm (mean, 93.6%, SD = 3.3%; *N* = 16; not shown), indicating that this region is also a paternal germline methylation imprint, and together indicating that the entire *IGF2/H19* imprinted domain may acquire the paternal methylation marks present in somatic tissues during spermatogenesis.

### CTCF binding within *IGF2* intron 3

**Figure 5:**
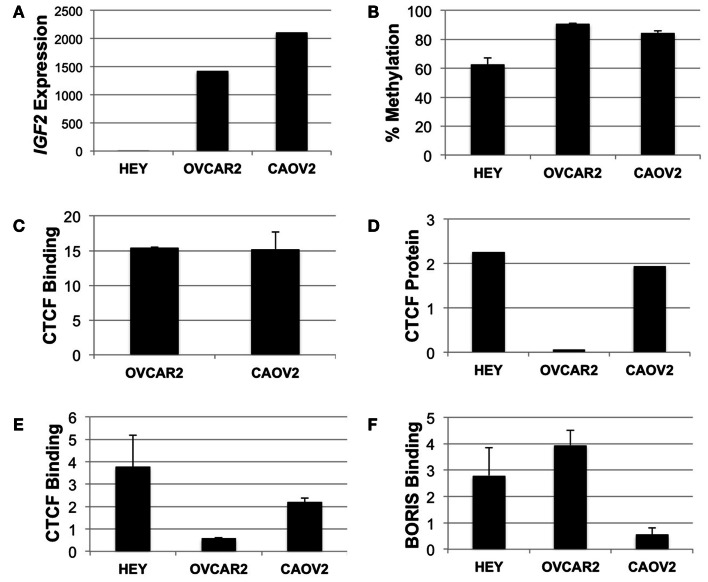
***IGF2* analyses in HEY, CAOV2, and OVCAR2 ovarian cancer cell lines**. **(A)** *IGF2* transcription levels in each cell line; **(B)** *IGF2* intron 3 region methylation levels; **(C)** ChIP assay for binding of CTCF to the *MYC* locus in CAOV2 and OVCAR2 cells (HEY cells were not analyzed); **(D)** Western blot detection of CTCF protein levels; **(E)** ChIP assay for binding of CTCF to the *IGF2* intron 3 CTCF binding motif; **(F)** ChIP assay for binding of BORIS to the *IGF2* intron 3 CTCF binding motif.

We performed mechanistic analyses of the intragenic CTCF binding site in three ovarian cancer cell lines, including HEY, OVCAR2, and CAOV2. HEY cells have the lowest *IGF2* transcript levels, as do many other established ovarian cancer cell lines (Matsumura et al., 2011), while the other two lines exhibit higher *IGF2* expression (Figure 5A). By pyrosequencing, methylation of the intron 3 region was lowest (66%) in the HEY cells, while the other lines showed >85% methylation at these CpG dinucleotides (Figure 5B).

Chromatin immunoprecipitation was used to determine if CTCF binds to the region containing the consensus CTCF binding motif. As a positive control, the anti-CTCF antibody was used to test for binding at the *MYC* locus, known to have high CTCF binding activity (Pugacheva et al., 2005). ChIP results showed enrichment of CTCF binding at the *MYC* CTCF binding site relative to the no antibody control (Figure 5C).

We next examined CTCF binding to the putative CTCF consensus binding sequence within *IGF2*. Although CAOV2 and OVCAR2 were obtained from different sources, our testing showed that they are genetically identical (Korch et al., 2012). Despite this, CAOV2 has much higher endogenous *CTCF* mRNA transcript levels (not shown) and protein expression as measured by Western blotting than OVCAR2 (Figure 5D), providing the unique opportunity to evaluate the role of differential CTCF expression in these otherwise genetically identical cells. OVCAR2 cells did not exhibit enriched binding of CTCF to the intron 3 CTCF binding motif relative to the control, while CAOV2 and HEY showed ∼2.2 to 3.8-fold enrichment of CTCF binding (Figure 5E).

### BORIS binding at the *IGF2* intron 3 CTCF site

We also determined if the BORIS protein (encoded by *CTCFL*) binds at the CTCF motif. BORIS and CTCF are paralogs that have the same binding specificity, with BORIS normally expressed during spermatogenesis. BORIS has been shown to become deregulated in cancers, and to compete with CTCF for binding occupancy (Klenova et al., 2002). Indeed, by ChIP, BORIS was enriched at the intron 3 CTCF site in HEY and OVCAR2 (in which CTCF does not bind) but BORIS was not enriched at the intron 3 CTCF site in CAOV2 cells (Figure 5F).

### Repressor/insulator activity of *IGF2* intron 3

We used a luciferase reporter assay to determine how the presence of the *IGF2* intron 3 CTCF binding motif influences transcriptional activity. We tested three constructs, including the vector alone, the vector containing the CTCF binding motif, and the vector containing an intronic sequence from within the *GAPDH* locus with similar nucleotide composition but that does not contain a consensus CTCF binding motif. Luciferase activity was normalized to that obtained for the pGL3 vector alone.

**Figure 6:**
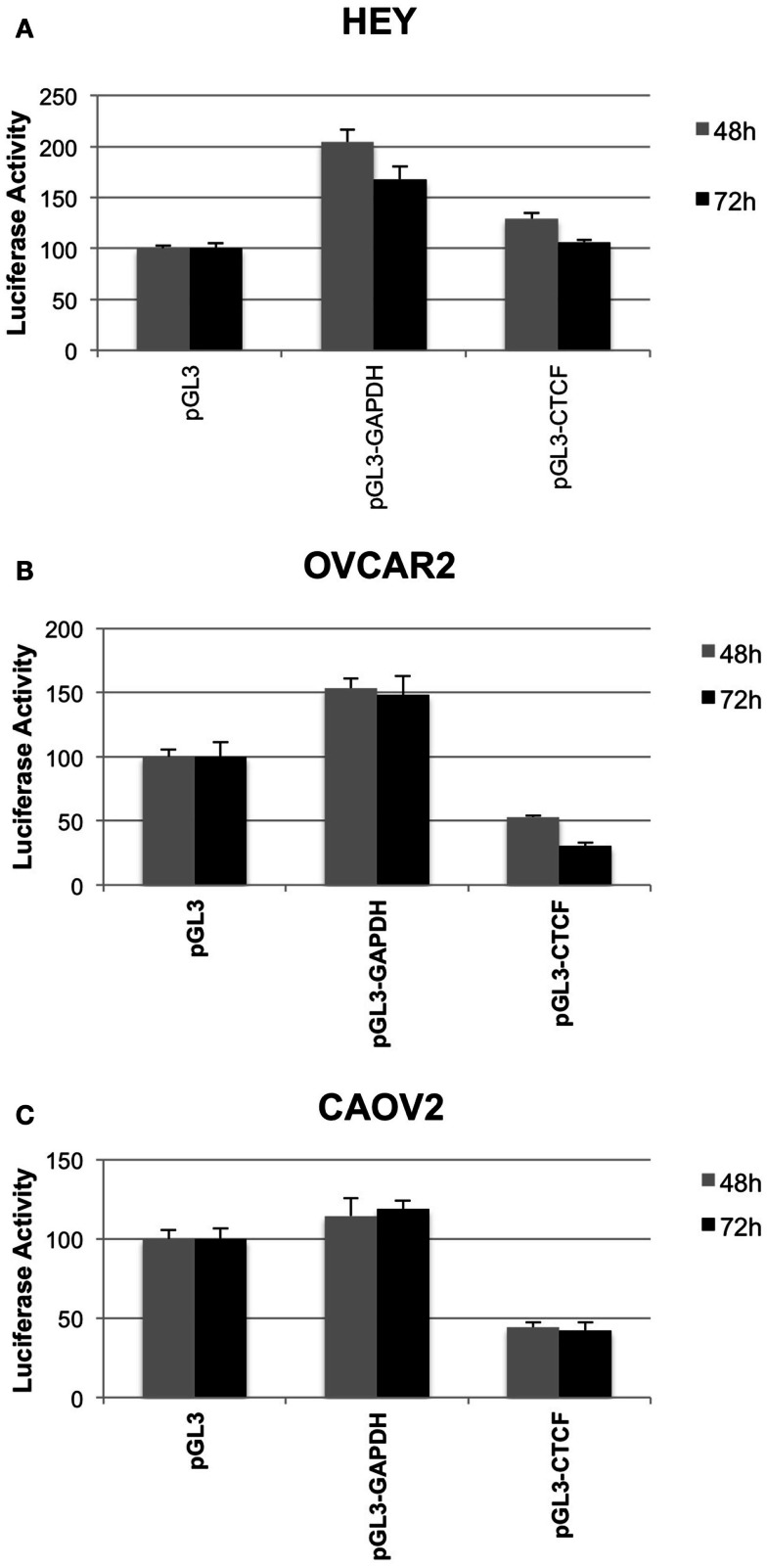
**Insulator activity of the *IGF2* intron 3 CTCF binding motif in ovarian cancer cells**. Luciferase activity from various pGL3 plasmid constructs, as a read out for the capacity of the *IGF2* intron 3 sequence motif to inhibit interactions between the enhancer and promoter of the plasmid, is shown on the *y* axis (+SD) in each panel. HEY cells **(A)**, OVCAR2 cells **(B)** and CAOV2 cells **(C)** were analyzed in sextuplicate at 48 h (light gray) and 72 h (dark gray) post-plasmid transfection. pGL3, control plasmid with no insert; pGL3-GAPDH, plasmid with sequence fragment of the *GAPDH* locus that does not contain a consensus CTCF binding motif; pGL3-CTCF, plasmid with the *IGF2* intron 3 CTCF binding motif.

In all three of the cell lines, the presence of the *GAPDH* sequence fragment was not inhibitory to transcription, with increased luciferase activity relative to the vector control (Figure 6). In the OVCAR2 and CAOV2 lines that have no or relatively low CTCF binding activity, respectively, the presence of the *IGF2* intron 3 CTCF binding region reduced luciferase activity, suggesting that this sequence element may function as an insulator or repressor in these cells. In the HEY cells, which have the highest level of CTCF binding activity, lowest *IGF2* transcription, and lowest methylation among the three lines tested, the CTCF region was not inhibitory to transcription.

## Discussion

We have identified a novel consensus binding sequence for the CTCF protein within an intron of *IGF2*. The *IGF2/H19* imprinted domain contains an intensively studied cluster of CTCF binding sites that are located upstream from the *H19* promoter region. Hypermethylation of the paternally derived allele and hypomethylation of the maternally derived allele at this ICR is required for the appropriate establishment of imprinted expression in mice, and CTCF binding to the hypomethylated maternally derived chromosome maintains the unmethylated state of this allele in somatic tissues (Schoenherr et al., 2003; Szabo et al., 2004). CTCF binding to the orthologous region in humans may not be as critical in regulating expression of *IGF2* or *H19* (Ulaner et al., 2003).

We had previously shown a positive correlation with methylation at the *IGF2/H19* ICR and *IGF2* expression that was not related to loss of imprinting (Murphy et al., 2006). In the present study, we also determined that there is a significant positive correlation between methylation of the intron 3 CpG sites and *IGF2* transcription in serous epithelial ovarian cancers. Our assay measured methylation of two CpG dinucleotides, including one within the CTCF consensus binding sequence and another located 13 bp away, just outside the consensus sequence. The CpG within the CTCF binding motif showed consistently higher methylation than the one positioned outside the binding motif in all tissues analyzed, but the significance of this observation is presently unclear. Regardless of the difference in methylation between the two sites within a given tissue, they both showed higher methylation in normal tissues and tumors with higher *IGF2* transcript levels, suggesting that this region may contribute to regulating *IGF2* activity.

A hallmark of imprinted genes is parental allele-specific methylation that is established during gametogenesis. Methylation at the *IGF2/H19* ICR is present on the paternally derived chromosome and is one of only several regions in the human genome known to contain a paternally derived gametic imprint mark. Most of the methylation associated with genomically imprinted genes is established on maternally derived chromosomes. We analyzed 16 independent human spermatozoa specimens to determine if the methylation at the intron 3 region of *IGF2* was established during spermatogenesis. The spermatozoa all showed very high methylation of these CpG sites, indicating that the methylation is established during spermatogenesis, and consistent with the idea that these CpG sites may also be a component of the gametic imprint mark for the *IGF2/H19* domain. Further supporting this notion was the finding that the *IGF2* DMR is also highly methylated in the spermatozoa, in agreement with the methylation findings in sperm for this region from another study of infertility (Boissonnas et al., 2010). In human conceptal tissues, we found that the intron 3 region had mean methylation values ranging from 39.1% (placenta) to 66.1% (heart). These findings suggest that this region may maintain parent-of-origin-dependent methylation in somatic tissues.

The consensus CTCF sites at the human ICR each harbor four CpG dinucleotides, while the novel CTCF site in *IGF2* intron 3 analyzed herein contains a single CpG dinucleotide. CTCF is known to bind to the ICR in a methylation-sensitive manner (Hark et al., 2000; Kanduri et al., 2000). CTCF binding induces a change in chromatin structure that results in insulator activity. Insulation at the ICR on the unmethylated maternal chromosome creates a blockade preventing interaction between the *IGF2* promoters from enhancers downstream of *H19*, thus contributing to *IGF2* repression on the maternal chromosome (Bell et al., 1999; Bell and Felsenfeld, 2000; Kanduri et al., 2000). This regulatory activity may also involve intrachromosomal interactions within the domain as well as interactions with matrix attachment regions (Kurukuti et al., 2006). The methylation status of the intron 3 CTCF consensus sequence and correlation with expression of *IGF2* in ovarian cancer prompted us to hypothesize that methylation status may modulate binding of CTCF and that this in turn is influencing transcriptional activity of *IGF2*. Our results suggest that methylation of the single CpG dinucleotide within the *IGF2* intron 3 CTCF binding motif may be inhibitory to CTCF binding.

The CTCF paralog, BORIS, has identical zinc fingers and DNA binding specificity as CTCF, but the sequences diverge outside the zinc fingers, conferring differences in regulatory capacities. BORIS was reported to not be detectable in most normal tissues due to exclusive expression of BORIS in the testis (Loukinov et al., 2002). More recently, however, others have reported that BORIS is indeed also expressed in normal tissues (Jones et al., 2011). Importantly, BORIS is abnormally activated in many different types of cancer (Klenova et al., 2002), including gynecologic malignancies (Risinger et al., 2007), and may compete with CTCF for binding sites in this context.

We found a dramatically different CTCF and BORIS binding activity at the consensus CTCF binding motif in intron 3 in genetically identical CAOV2 and OVCAR2 cells, both of which express relatively high levels of *IGF2*. The highest level of BORIS binding was detected in OVCAR2 cells, which have very low CTCF protein levels and do not exhibit detectable CTCF occupancy of the same site (compare Figure 5F to Figures 5D,E). Conversely, the lowest level of BORIS binding was in the CAOV2 cells, which are enriched for CTCF binding. The HEY cells, with virtually no expression of *IGF2* mRNA and the lowest methylation of the intron 3 region, showed enrichment of both CTCF and BORIS binding. Our results demonstrate that CTCF and BORIS are both able to bind the *IGF2* intron 3 CTCF binding motif and that this binding is associated with differences in methylation and expression.

Results from a luciferase reporter assay showed that the presence of the *IGF2* intron 3 CTCF binding motif had no effect on luciferase transcription in HEY cells, while it was inhibitory to transcription in both OVCAR2 and CAOV2 cells. Taken together, our results are perhaps most parsimonious with the idea that methylation of the intron 3 CTCF binding motif is inhibitory to CTCF/BORIS binding at the endogenous locus but permissive for binding in the context of the (presumed unmethylated) reporter construct. Furthermore, these results suggest that this region indeed has insulator function when unmethylated and bound to CTCF or BORIS, as is the case for the reporter construct in the CAOV2 and OVCAR2 cells, respectively. In the HEY cells, CTCF and BORIS may compete with each other for binding the intron 3 CTCF binding motif in the reporter construct with a net null outcome. These results contrast with those observed at the endogenous locus in these same cells, which differ at least in part by methylation status. The CAOV2 and OVCAR2 cells have high methylation at the intron 3 CTCF binding motif and express *IGF2* at high levels, indicative of at least partial inhibition of CTCF or BORIS binding and low insulator activity function. In contrast, HEY cells have lower methylation, do not express *IGF2* and exhibit binding of both CTCF and BORIS to the intron 3 CTCF binding motif. These results support the idea that lower levels of methylation are permissive for CTCF/BORIS binding, which leads to insulation of the *IGF2* promoter(s) from enhancer activity. This is in agreement with the findings *in vivo*, where high levels of methylation at the intron 3 CTCF binding motif are correlated with higher levels of *IGF2* transcription in both normal and malignant tissues. Indeed, mouse studies have shown that paternal methylation at an intragenic differentially methylated region of *Igf2* (referred to as DMR2) is associated with increased transcription (Murrell et al., 2001). However, unlike the human *IGF2* intron 3 CTCF binding motif analyzed here, the core *Igf2* DMR2 region in the mouse is located within an exon and is densely populated with CpGs dinucleotides.

A limitation of our study is that we did not measure the binding of CTCF or BORIS within the reporter construct, so we do not know if the results obtained are indeed consequent to binding of the endogenous proteins to the reporter construct. Furthermore, it was not possible to directly test how methylation of the CpG sites within and adjacent to the intron 3 CTCF motif inserted into the reporter construct might have influenced protein binding and luciferase activity. It is possible that binding of these proteins at the endogenous locus in CAOV2 or OVCAR2 occurs only on the 10–15% of alleles within the cell population lacking methylation. Our results show enrichment of binding of CTCF or BORIS in CAOV2 and OVCAR2 cells, respectively, but do not address what proportion of the alleles are actually bound by these proteins.

In conclusion, elevated *IGF2* expression in epithelial ovarian cancers is associated with increased methylation of a novel regulatory sequence motif that we have shown binds to the CTCF protein. The growth-stimulatory function of IGF2 likely confers a substantial advantage to malignant cells, given the ubiquity of *IGF2* overexpression in ovarian cancer. Elucidation of the mechanisms that control *IGF2* transcription is necessary if we hope to target this gene therapeutically. Positive correlation between methylation and *IGF2* transcription in primary ovarian cancers suggests the potential for epigenetic therapies in controlling *IGF2* overexpression in this disease.

## Conflict of Interest Statement

The authors declare that the research was conducted in the absence of any commercial or financial relationships that could be construed as a potential conflict of interest.
